# Physical Fitness, White Matter Volume and Academic Performance in Children: Findings From the ActiveBrains and FITKids2 Projects

**DOI:** 10.3389/fpsyg.2019.00208

**Published:** 2019-02-12

**Authors:** Irene Esteban-Cornejo, Maria Rodriguez-Ayllon, Juan Verdejo-Roman, Cristina Cadenas-Sanchez, Jose Mora-Gonzalez, Laura Chaddock-Heyman, Lauren B. Raine, Chelsea M. Stillman, Arthur F. Kramer, Kirk I. Erickson, Andrés Catena, Francisco B. Ortega, Charles H. Hillman

**Affiliations:** ^1^Center for Cognitive and Brain Health, Department of Psychology, Northeastern University, Boston, MA, United States; ^2^PROFITH “PROmoting FITness and Health through Physical Activity” Research Group, Department of Physical Education and Sports, Faculty of Sport Sciences, University of Granada, Granada, Spain; ^3^Department of Experimental Psychology, Mind, Brain and Behavior Research Center (CIMCYC), University of Granada, Granada, Spain; ^4^Beckman Institute, University of Illinois at Urbana-Champaign, Urbana, IL, United States; ^5^Department of Psychiatry, University of Pittsburgh, Pittsburgh, PA, United States; ^6^Brain Aging and Cognitive Health Lab, Department of Psychology, University of Pittsburgh, Pittsburgh, PA, United States; ^7^Department of Physical Therapy, Movement, and Rehabilitation Sciences, Northeastern University, Boston, MA, United States

**Keywords:** aerobic capacity, motor ability, speed-agility, muscular strength, brain structure, academic achievement, obesity, childhood

## Abstract

**Objectives:** The aims of this study were (i) to examine the association between cardiorespiratory fitness and white matter volume and test whether those associations differ between normal-weight and overweight/obese children (ii) to analyze the association between other physical fitness components (i.e., motor and muscular) and white matter volume, and (iii) to examine whether the fitness-related associations in white matter volume were related to academic performance.

**Methods:** Data came from two independent projects: ActiveBrains project (*n* = 100; 10.0 ± 1.1 years; 100% overweight/obese; Spain) and FITKids2 project (*n* = 242; 8.6 ± 0.5 years; 36% overweight/obese, United States). Cardiorespiratory fitness was assessed in both projects, and motor and muscular fitness were assessed in the ActiveBrains project. T1-weighted images were acquired with a 3.0 T S Magnetom Tim Trio system. Academic performance was assessed by standardized tests.

**Results:** Cardiorespiratory fitness was associated with greater white matter volume in the ActiveBrain project (*P* < 0.001, *k* = 177; inferior fronto-opercular gyrus and inferior temporal gyrus) and in the FITKids project (*P* < 0.001, *k* = 117; inferior temporal gyrus, cingulate gyrus, middle occipital gyrus and fusiform gyrus) among overweight/obese children. However, no associations were found among normal-weight children in the FITKids project. In the ActiveBrains project, motor fitness was related to greater white matter volume (*P* < 0.001, *k* = 173) in six regions, specifically, insular cortex, caudate, bilateral superior temporal gyrus and bilateral supramarginal gyrus; muscular fitness was associated with greater white matter volumes (*P* < 0.001, *k* = 191) in two regions, particularly, the bilateral caudate and bilateral cerebellum IX. The white matter volume of six of these regions were related to academic performance, but after correcting for multiple comparisons, only the insular cortex remained significantly related to math calculations skills (β = 0.258; *P* < 0.005). In both projects, no brain regions showed a statistically significant negative association between any physical fitness component and white matter volume.

**Conclusion:** Cardiorespiratory fitness may positively relate to white matter volume in overweight/obese children, and in turn, academic performance. In addition, motor and muscular fitness may also influence white matter volume coupled with better academic performance. From a public health perspective, implementing exercise interventions that combine aerobic, motor and muscular training to enhance physical fitness may benefit brain development and academic success.

## Introduction

The brain undergoes significant changes during childhood (Giedd et al., 1999). Further, aspects of cognition, including academic performance, continue to develop throughout the school-aged years. This period of neurodevelopment may be particularly sensitive to health-related factors that influence brain and behavior (Andersen, 2003; Donnelly et al., 2016). In particular, physical fitness is a powerful marker of health that has been associated with brain structure and function, as well as cognition in children (Ortega et al., 2008b; Voss et al., 2011; Esteban-Cornejo et al., 2014; Donnelly et al., 2016). The three main components of physical fitness are cardiorespiratory, motor and muscular fitness, each of them may have different influences on the brain. Cardiorespiratory fitness is the capacity to carry out prolonged strenuous exercise; motor fitness is a combination of speed, agility and coordination, and muscular fitness is the capacity to carry out work against a resistance (Ortega et al., 2008b). Specifically, we have previously shown that cardiorespiratory fitness is associated with greater gray matter volume of the hippocampus and the basal ganglia in both normal–weight and overweight/obese children (Chaddock et al., 2010a,b; Esteban-Cornejo et al., 2017). Additionally, cardiorespiratory fitness and motor fitness, but not muscular fitness, were associated with greater gray matter volume in distinct cortical regions (i.e., frontal, temporal and calcarine cortices) in overweight/obese children (Esteban-Cornejo et al., 2017). In turn, these brain-related associations were coupled with better executive function and academic performance (Chaddock et al., 2010a; Esteban-Cornejo et al., 2017). However, less is known about how the different components of physical fitness (i.e., cardiorespiratory, motor and muscular) may influence white matter tissue, and in turn, academic performance during childhood.

White matter is primarily comprised of glial cells and myelinated neurons. White matter growth is the main source of increased brain volume during child development and continues well into the second decade of life for some brain regions. While cortical gray matter seems to develop in a non-linear trend, with a preadolescent increase followed by a postadolescent decrease, white matter follows a linear trend and continues to mature during childhood and adolescence, increasing its volume and becoming more myelinated (Giedd et al., 1999; Paus et al., 2001). Damage to white matter yields slower processing speed (Kail, 1998), which may impair academic performance (van Eimeren et al., 2008). To date, only two previous studies in youth have examined the association between cardiorespiratory fitness and structure of white matter (Chaddock-Heyman et al., 2014; Herting et al., 2014). Specifically, cardiorespiratory fitness was positively related to the microstructure of white matter fiber tracts (i.e., corpus callosum, corona radiata, and longitudinal fasciculus) in children (Chaddock-Heyman et al., 2014); whereas among adolescents, cardiorespiratory fitness was negatively related to white matter microstructure in the corticospinal tract (Herting et al., 2014). As such, it is difficult to draw a conclusion from those studies due to their contradictory findings. In addition, other dimensions of fitness (i.e., muscular and motor fitness) may have differential effects on white matter, and in turn, academic performance, similar to previous reports in relation to gray matter (Esteban-Cornejo et al., 2017). Lastly, those studies were mainly focused on normal-weight populations; however, obesity has also been associated with alterations in white matter volume and integrity as compared to normal-weight individuals (Kullmann et al., 2015; van Bloemendaal et al., 2016). Indeed, the brain’s volumetric structure of individuals with overweight and obesity is 10 years older compared to that of their lean peers, pointing to accelerated aging of white matter structure in overweight/obese (Ronan et al., 2016). For example, obese children have shown white matter reduction in the cerebellar peduncles and lower academic performance than their normal-weight peers (Augustijn et al., 2017; Raine et al., 2018). Therefore, there is a clear need for studies that examine the different components of fitness and their associations with white matter volume in both normal-weight and overweight/obese populations, as well as their coupled influence on academic performance to better determine the relation of health factors on brain structure and cognition during child development.

We have a unique opportunity to test these hypotheses using baseline data from two independently, relatively large, trials conducted on children in Spain and the United States: the ActiveBrains project (Spain) which includes overweight/obese children and the FITKids2 project (United States) which includes both normal-weight and overweight/obese children. As such, our main aim was to examine the association between cardiorespiratory fitness and white matter volume in these two similarly designed, yet independent, studies to better determine the consistency of the relationship between this aspect of fitness and white matter. In addition, we examined whether the abovementioned associations differ between normal-weight and overweight/obese children using data from the FITKids2 project and we analyzed the association between other physical fitness components (i.e., motor and muscular) and white matter volume using data from the ActiveBrains project. To achieve these aims, we performed whole-brain exploratory analyses because, to date, there is no a substantial body of evidence on the associations between physical fitness components and white matter in children. Lastly, we examined whether the fitness-related associations in white matter volume were related to academic performance across these two independent studies.

## Materials and Methods

### Participants

The ActiveBrains and FTIKids2 projects are randomized controlled trials designed to examine the effects of an exercise program on brain, cognition and academic performance in children aged 7–11 years. For the ActiveBrains project^1^, a total of 110 overweight/obese children aged 8–11 years were recruited from schools in Granada, Spain (Cadenas-Sanchez et al., 2016). Eligible children were required to: (1) be overweight or obese based on World Obesity Federation cut-off points (2) be 8–11 years-old, (3) not have any physical disabilities or neurological disorder that affects their physical performance, and (4) in the case of girls, not to have started the menstruation at the moment of the assessments. Baseline data were collected from November 2014 to February 2016. Parents or legal guardians were informed of the purpose of the study and written informed parental and child consents were obtained. The ActiveBrains project was approved by the Human Research Ethics Committee of the University of Granada, and was registered in ClinicalTrials.gov (identifier: NCT02295072).

For the FITKids2 project, a total of 252 children aged 7-9 years were recruited from schools in East-Central Illinois, United States. Eligible children were required to (1) report an absence of school related learning disabilities (i.e., individual education plan related learning), adverse health conditions, physical incapacities, or neurological disorders, (2) qualify as prepubescent (Tanner pubertal timing score ≤ 2), (3) report no use of medications that influence central nervous system function, and (4) demonstrate right handedness as measured by the Edinburgh Handedness Questionnaire. Data were collected from June 2010 to October 2017. Children signed an informed assent and parents or legal guardians provided written informed consent in accordance with the Institutional Review Board of the University of Illinois at Urbana-Champaign, and was registered in ClinicalTrials.gov (identifier: NCT01619826).

For the present study, we selected children from the ActiveBrains and FITKids2 projects with complete baseline data on physical fitness, academic performance and brain outcomes (i.e., white matter volume). A total of 100 overweight/obese children from the ActiveBrains project (10.0 ± 1.1 years; 40% girls) met all the criteria. A total of 142 children from the FITKids2 project (8.6 ± 0.5 years; 54% girls; 36% overweight/obese) met all the criteria; FITKids2 children included in the present study did not differ from those not included across measures of height, weight, peak height velocity (PHV), body mass index (BMI), parental education, cardiorespiratory fitness, total white matter and academic performance data (all *p* > 0.05). The present study includes a total of 242 children.

### Physical Fitness

In the ActiveBrains project, physical fitness was assessed following the ALPHA (Assessing Levels of Physical fitness and Health in Adolescents) health-related fitness test battery for youth, a feasible, reliable and valid battery for this age group (Ortega et al., 2008a; Castro-Pinero et al., 2010a; Ruiz et al., 2011). All tests were performed in a single session. The three main physical fitness components were assessed: cardiorespiratory, motor and muscular fitness.

*Cardiorespiratory fitness* was assessed by the 20-m shuttle-run test. The test was performed once and always at the end of the fitness testing session. Participants were required to run between two lines 20-m apart, while keeping pace with a pre-recorded audio CD. The initial speed was 8.5 km/h, which was increased by 0.5 km/h each minute (1 min = 1 stage). Participants were instructed to run in a straight line, to pivot on completing a shuttle (20-m), and to pace themselves in accordance with the audio signals. The test was finished when the participant failed to reach the end lines concurrently with the audio signals on two consecutive occasions. The last stage completed was recorded and transformed to maximal oxygen consumption (VO_2_max, mL/kg/min) using the Léger equation (Leger et al., 1988).

*Motor fitness* was assessed with the 4 × 10-m shuttle-run test of speed-of-movement, agility and coordination. The test was performed twice and the fastest time was recorded in seconds (Vicente-Rodriguez et al., 2011). Participants were required to run back and forth twice between two lines 10-m apart. Children were instructed to run as fast as possible and every time they crossed any of the lines, they were instructed to pick up (the first time) or exchange (second and third time) a sponge that had earlier been placed behind the lines. Since a longer time indicates poorer performance (i.e., the person is slower and less agile and coordinated), the variable expressed in seconds was inverted by multiplying by -1, so that a higher score indicates better performance.

*Muscular fitness* was assessed using maximum handgrip strength and the standing long jump tests (Artero et al., 2012). A hand dynamometer with an adjustable grip was used (TKK 5101 Grip D, Takey, Tokyo Japan) for the handgrip strength test. The participant squeezed the dynamometer continuously for at least 2-s, alternatively with right and left hand, with the elbow in full extension (Espana-Romero et al., 2010). The test was performed twice and the maximum score for each hand was recorded in kilograms (kg). The average score of the left and right hands was calculated in kg as an absolute measurement of upper body muscular fitness (Espana-Romero et al., 2008; Espana-Romero et al., 2010). Standing long jump test was performed from a starting position behind a line, standing with feet approximately shoulder width apart (Castro-Pinero et al., 2010b). Children jumped as far forward as possible, landing with feet together. The test was performed three times. The longest distance was recorded in centimeters, and subsequently multiplied by body weight in order to obtain an absolute measurement of lower body muscular fitness. A single muscular fitness score was computed from the two muscular tests. The individual score of each test was standardized as follows: *Z*-standardized value = (value – the sample mean)/SD. The muscular fitness score was calculated as the mean of the two standardized scores.

In the FITKids2 project, only cardiorespiratory fitness was assessed. It was determined by measuring VO_2max_ using a computerized indirect calorimetry system (Parvo Medics True Max 2400, Sandy, UT, United States) during a modified Balke Protocol. Children walked and/or ran on a treadmill at a constant speed with increasing grade increments of 2.5% every 2 min until volitional exhaustion occurred (American College of Sports Medicine, 2014). Average for oxygen consumption and respiratory exchange ratio (RER) assessed every 20 s. A polar heart rate monitor (Polar WearLink+ 31; Polar Electro, Finland) was used to measure heart rate throughout the test, and ratings of perceived exertion (RPE) were assessed every 2 min using the children’s OMNI (Utter et al., 2002). VO_2max_ was expressed in mL/kg/min and based upon maximal effort as evidenced by (i) a plateau in oxygen consumption corresponding to an increase of less than 2 mL/kg/min despite an increase in workload; (ii) a peak heart rate ≥ 185 beats per minute (American College of Sports Medicine, 2014) and heart rate plateau (Freedson and Goodman, 1993); (iii) RER ≥ 1.0 (Bar-Or, 1983); and/or (iv) a score on the children’s OMNI ratings of perceived exertion (RPE) scale ≥ 8 (Utter et al., 2002).

### Magnetic Resonance Imaging (MRI) Procedure

#### Data Acquisition

Data were collected using a 3.0 Tesla Siemens Magnetom Tim Trio system (Siemens Medical Solutions, Erlangen, Germany) with a 32-channel head coil in the ActiveBrains project and a 3.0 Tesla Siemens Magnetom Tim Trio system (Siemens Medical Solutions, Erlangen, Germany) with a 12-channel head coil in the FITKids2 project. Three-dimensional, high-resolution, T1-weighted images were acquired using a magnetization-prepared rapid gradient-echo (MPRAGE) sequence. In the ActiveBrains project, the parameters were as follows: repetition time (TR) = 2300 ms, echo time (TE) = 3.1 ms, inversion time (TI) = 900 ms, flip angle = 9°, Field of view (FOV) = 256 × 256, acquisition matrix = 320 × 320, 208 slices, resolution = 0.8 × 0.8 × 0.8 mm, and scan duration of 6 min and 34 s. In the FITKids2 project, the parameters were as follows: TR = 1900 ms, TE = 2.32 ms, TI = 900 ms, flip angle = 9°, FOV = 230 × 230 acquisition matrix = 256 × 256, 192 slices, resolution = 0.9 × 0.9 × 0.9 mm, and scan duration of 4 min and 26 s.

#### Structural Image Processing

Structural imaging data were pre-processed using Statistical Parametric Mapping software (SPM12; Wellcome Department of Cognitive Neurology, London, United Kingdom) implemented in Matlab (The MathWorks, Inc., Natick, MA, United States). Before tissue classification we checked each individual image for acquisition artifacts and alignment along the horizontal anterior commissure and posterior commissure plane.

Detailed information about the pre-processing steps is available elsewhere and is outlined briefly in this section (Esteban-Cornejo et al., 2017). First, T1-weighted structural images of each participant were segmented into gray matter tissue, white matter tissue, and cerebrospinal fluid using the segmentation algorithm implemented in SPM12 (Ashburner and Friston, 2005). Second, we used segmented gray matter/white matter tissues for all participants to create a customized template using Diffeomorphic Anatomical Registration Through Exponentiated Lie algebra (DARTEL) (Ashburner, 2007). DARTEL estimates a best set of smooth deformations from every participant’s tissue to their common average and reiterates the process until convergence. The resultant images were spatially normalized to Montreal Neurological Institute (MNI) space with affine transformation to create the DARTEL template. We create a DARTEL template for the ActiveBrains project and a DARTEL template for the FITKids2 project. Subsequently, we normalized each participant’s segmented images in each study to each specific DARTEL template via non-linear transformation. To perform a volume change correction, the normalized images were modulated with Jacobian determinants derived from the spatial normalization (Ashburner and Friston, 2000). Finally, the volumetric images were smoothed by convolving them with an isotropic Gaussian kernel of 8 mm full-width at half-maximum (FWHM).

### Academic Performance

In the ActiveBrains project, academic performance was assessed via the Spanish version of the Woodcock-Johnson III (i.e., Bateria III Woodcock-Muñoz, pruebas de aprovechamiento). This battery is a well validated measure of academic performance from individuals aged 5–95 years (McGrew and Woodcock, 2001). We applied 12 tests: 11 from the standard battery (i.e., 3 tests of reading, 3 tests of mathematics, 2 tests of oral language and 3 tests of written language) and one from the extended battery (i.e., a test based on science, social science and humanities). All the tests were individually administered by a trained evaluator in one session of 100–120 min. The data collected for each participant was independently checked by two trained evaluators. In the FITKids2 project, participants completed the Kaufman Test of Educational Abilities (KTEA2) to assess academic performance. Subtests of the KTEA2 were administered to assess their achievements in the content areas of mathematics, reading, and writing. The KTEA2 was individually administrated by a trained evaluator in one session of 60–80 min. The main dependent measures in both projects were the standard scores of 6 academic indicators: mathematics, mathematics calculation skills, reading, writing, written expression and total achievement. Science was also included as an additional academic indicator in the ActiveBrians project.

### Covariates

#### Body Mass Index

Body weight and height were performed with participants having bare feet and wearing underclothes; weight was measured with an electronic scale (ActiveBrains: SECA 861, Hamburg, Germany; FITKids2: Tanita WB-300 Plus digital scale, Tokyo, Japan) and height (cm) with a stadiometer (ActiveBrains: SECA 225, Hamburg, Germany; FITKids2: SECA 240, Hamburg, Germany). Both measurements were performed twice in the ActiveBrains project and three times in the FITIKids2 project in the same session, and averages were used. BMI was expressed as kg/m^2^ and children were categorized as normal-weight, overweight and obesity according to Cole and Lobstein (2012).

#### Biological Maturation

Peak height velocity is a common indicator of maturity in children and adolescents and it used as a maturational landmark due to its relevance in previous studies (Malina et al., 2015). In both projects, PHV was obtained from anthropometric variables (weight, height, and/or seated height) using Moore’s equations through validated sex-specific algorithms for children (Moore et al., 2015). Years from PHV were calculated by subtracting the age of PHV from the chronological age. The difference in years was defined as a value of maturity offset.

#### Parental Education Level

In both projects, socioeconomic status was assessed by the educational level of the mother and father reported as none, elementary school, middle school, high school and university completed. Parent responses were combined as: none of the parents with university studies, one of them had university studies and both had university studies (Huppertz et al., 2016).

### Statistical Analysis

All the analyses were performed separately for the ActiveBrains project and the FITKids2 project. In the ActiveBrains project, the analyses were performed for the whole overweight/obese sample together; in the FITKids2 project, the analyses were performed separately for normal-weight and overweight/obese children. Descriptive statistics are presented as means (SD) or percentages using IBM SPSS Statistics (version 18.0 for Windows; *P* set at < 0.05).

Statistical analyses of imaging data were performed using the GLM approach implemented in SPM12. The individual association between each component of physical fitness (i.e., cardiorespiratory, motor and muscular in the ActiveBrains project and cardiorespiratory in the FITKids2 project) and white matter volume was analyzed using whole-brain voxelwise multiple regression models, adjusted for sex, PHV offset, parent education, and BMI. Additionally, we extracted the eigenvalues from the peak coordinates of each significant cluster. The associations of the extracted mean white matter volumes as predictor variables and academic performance indicators as outcomes, adjusted for sex, PHV offset, parental education and BMI were examined by linear regressions in SPSS. We corrected for assessing multiple white matter-academic performance regressions by defining statistical significance as a Benjamini-Hochberg False Discovery Rate q less than 0.05 (Benjamini and Hochberg, 1995).

The statistical threshold in the imaging analyses was calculated with AlphaSim, as implemented in Resting-State fMRI Data Analysis Toolkit toolbox (RESTplus) (Song et al., 2011). Parameters were defined as follows: cluster connection radius (rmm) = 5 mm and the actual smoothness of the data after model estimation, incorporating a white mask volume of 302567 voxels. The voxel-level alpha significance (threshold, *p* < 0.001 uncorrected) along with the appropriate cluster size for controlling for multiple comparisons in each analysis were indicated in the results. The resulting cluster extents were further adjusted to account for the non-isotropic smoothness of structural images, in accordance with Hayasaka et al. (2004).

## Results

### Background Characteristics

Table 1 shows the characteristics of the study sample from the ActiveBrains and the FITKids2 projects. The percentage of both parents having completed university studies was 16% in the ActiveBrains Project, 34% in overweight/obese FITKids2 children, and 48% in normal-weight FITKids2 children. In the ActiveBrains project, all participants were overweight/obese (26% overweight children from the total overweight/obese sample). In the FITKids2 project, 64% were normal-weight, 17% were overweight and 19% were obese. BMI was higher in overweigh/obese children from the ActiveBrains project (26.7 ± 3.7 kg/m^2^) relative to their overweight/obese peers from the FITKids2 project (22.5 ± 3.4 kg/m^2^). Cardiorespiratory fitness levels were higher for the normal-weight FITKids2 children (45.4 ± 6.7 mL/kg/min) than for the overweight/obese FITKids2 children (37.7 ± 5.6 mL/kg/min) and the ActiveBrains children (40.8 ± 2.8 mL/kg/min).

**Table 1:** Characteristics of samples from the ActiveBrains and FITKids2 projects.

	ActiveBrains (n = 100)	FITKids2 (n = 142)	
	Overweight/ obese	Normal-weight	Overweight/ obese
*n*	100	91	51
**Physical characteristics**			
Girls (%)	40	54	55
Age (years)	10.0 ± 1.1	8.6 ± 0.6	8.7 ± 0.5
Peak height velocity offset (years)	-2.3 ± 1.0	-3.3 ± 0.6	-3.0 ± 0.7
Weight (kg)	55.8 ± 11.0	28.7 ± 4.0	43.8 ± 8.8
Height (cm)	143.9 ± 8.3	133.0 ± 5.8	139.0 ± 7.1
Body mass index (kg/m^2^)	26.7 ± 3.7	16.2 ± 1.4	22.5 ± 3.4
Overweight (%)	26	–	47
**Parental education university level (%)**			
Neither parent	66	26	33
One parent	18	26	33
Both parents	16	48	34
**Physical fitness components**			
Cardiorespiratory fitness (mL/kg/min)^∗^	40.8 ± 2.8	45.4 ± 6.7	37.7 ± 5.6
Motor fitness (s)^†^	15.1 ± 1.6	–	–
Muscular fitness (*z*-score) ‡	0.0 ± 0.9	–	–
Total brain volume (cm^3^)	1200.3 ± 106.7	1199.3 ± 107.4	1217.9 ± 105.5
**Academic performance^∗∗^**			
Mathematics	101.7 ± 10.6	109.8 ± 16.5	105.3 ± 13.6
Math calculation skills	103.4 ± 12.0	107.4 ± 16.1	102.6 ± 13.2
Reading	108.2 ± 13.0	112.8 ± 14.6	107.6 ± 15.3
Writing	113.6 ± 12.7	105.6 ± 15.5	105.0 ± 15.6
Written expression	103.4 ± 8.7	102.2 ± 17.6	102.3 ± 17.5
Science	96.6 ± 11.5	–	–
Total achievement	109.1 ± 11.8	110.4 ± 14.7	106.4 ± 14.9


*Values are mean ± SD or percentages. ^∗^Measured by the 20-m shuttle run test in the ActiveBrains project and by the treadmill test in the FITKids2 project. ^†^Measured by the 4 × 10-m shuttle run test; values were multiplied by -1 before analyses so that higher values indicate better performance. ^‡^z-score computed from handgrip strength (kg) and standing long jump (cm^∗^kg) tests. ^∗∗^Assessed by the Spanish version of the Woodcock-Johnson III in the ActiveBrains project and by the Kaufman Test of Educational Abilities in the FITKids2 project.*

### White Matter Correlates of Individual Physical Fitness Components

**FIGURE 1:**
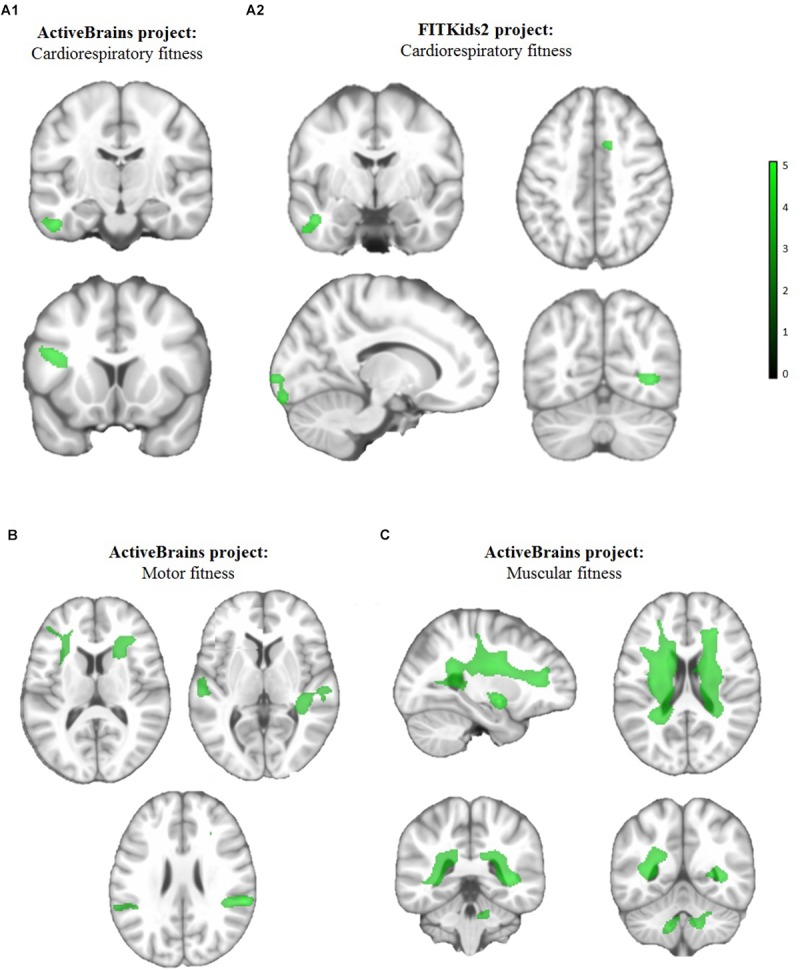
Brain regions showing positive separate associations of **(A1, A2)** cardiorespiratory fitness, **(B)** motor fitness and **(C)** muscular fitness with white matter volume in children from the ActiveBrains **(A1,B,C)** and FITKids2 **(A2)** projects. Analyses were adjusted by sex, peak height velocity offset (years), parent education university level (neither/one/both) and body mass index (kg/m^2^). Each physical fitness component was introduced in separate models. Maps were thresholded using AlphaSim at *P* < 0.001 with *k* = 177 voxels for cardiorespiratory fitness in the ActiveBrains project and *k* = 117 voxels in the FITKids2 project, *k* = 173 for motor fitness, and *k* = 191 for muscular fitness, and surpassed Hayasaka correction (see Table 2). The color bar represents *T*-values, with lighter green color indicating higher significant association. Images are displayed in neurological convention, whereby the right hemisphere corresponds to the right side in coronal displays. Sagittal planes show the left hemisphere.

Table 2 and Figure 1 present the brain regions showing positive associations between each of the components of physical fitness and white matter volume in children, after adjustment for potential confounders.

**Table 2:** Brain regions showing separate positive associations of the components of physical fitness with white matter volume in children from the ActiveBrains and FITKids2 projects.

	ActiveBrains (*n* = 100)					FITKids2 (*n* = 142)				
Brain Regions (mm^3^)	*X*	*Y*	*Z*	Peak *t*	Cluster size	*X*	*Y*	*Z*	Peak *t*	Cluster size
Normal-weight (*n* = 91)	*n* = 0					*n* = 91				
	No available data					Non-significant regions				
Overweight/obese(*n* = 151)	*n* = 100					*n* = 51				
**Cardiorespiratory fitness (mL/kg/min)^∗^**										
Inferior temporal gyrus	-53	-12	-33	4.1	312	-53	-6	-32	4.6	357
Inferior fronto-opercular gyrus	-44	17	23	4.2	552	–	–	–	–	–
Cingulate gyrus	–	–	–	–	–	12	9	44	4	138
Middle Occipital Gyrus	–	–	–	–	–	39	-66	2	4.4	342
Fusiform Gyrus	–	–	–	–	–	-26	-92	-9	4.5	951
**Motor fitness (s^-1^)^†^**										
Insular cortex	-30.0	32.0	8.0	3.7	1459	No available motor fitness data				
Caudate	33.0	30.0	12.0	3.9	1327					
Superior temporal gyrus	39.0	-32.0	5.0	3.9	1407					
Superior temporal gyrus	-56.0	-21.0	3.0	4.1	510					
Supramarginal gyrus	47.0	-38.0	29.0	5	768					
Supramarginal gyrus	-50.0	-41.0	26.0	3.6	216					
**Muscular fitness (z-score)‡**										
Caudate	24	26	11	4.4	13261	No available muscular fitness data				
Caudate	-12	11	-15	4.7	17512					
Cerebellum IX	14	-50	-42	3.9	755					
Cerebellum IX	-11	-51	-42	4.2	486					


*Analyses were adjusted by sex, peak height velocity offset (years), parent education university level (neither/one/both) and body mass index (kg/m^2^). Each physical fitness component was introduced in separate models. All contrasts were thresholded using AlphaSim at P < 0.001with k = 177 voxels for cardiorespiratory fitness in the ActiveBrains project and k = 117 voxels in the FITKids2 project, k = 173 for speed-agility and k = 191 for muscular fitness, and surpassed Hayasaka correction. Anatomical coordinates (X,Y,Z) are given in Montreal Neurological Institute (MNI) Atlas space. ^∗^Estimated by the Leger equation in the ActiveBrains project and directly measured by the treadmill test in the FITKids2 study. ^†^The original score of the motor fitness test expressed in seconds was multiplied by -1 to invert the variable, so that a higher score indicate higher fitness performance. ^‡^z-score computed from handgrip strength and standing long jump tests.*

In the ActiveBrains project, cardiorespiratory fitness (Figure 1A1) was associated with greater white matter volume (*P* < 0.001, *k* = 177) in two regions, inferior fronto-opercular gyrus and inferior temporal gyrus; motor fitness (Figure 1B) was related to greater white matter volume (*P* < 0.001, *k* = 173) in six regions, specifically, insular cortex, caudate, bilateral superior temporal gyrus and bilateral supramarginal gyrus; muscular fitness (Figure 1C) was associated with greater white matter volumes (*P* < 0.001, *k* = 191) in two regions, particularly, the bilateral caudate and bilateral cerebellum IX. In the FITKids2 project, among overweight/obese children, cardiorespiratory fitness (Figure 1A2) was associated with greater white matter volumes (*P* < 0.001, *k* = 117) in four regions, namely inferior temporal gyrus, cingulate gyrus, middle occipital gyrus and fusiform gyrus; whereas, no brain regions showed a statistically significant positive association between cardiorespiratory fitness and white matter volume among normal-weight children from the FITKids2 project. In both projects, no brain regions showed a statistically significant negative association between any physical fitness component and white matter volume.

### Association Between White Matter and Academic Performance

Table 3 displays the associations between fitness-related associations in white matter volume and academic performance, after controlling for potential confounders.

**Table 3:** Fitness-related associations in white matter volume and academic performance^†^ in overweight/obese children from the ActiveBrains and FITKids2 projects.

Fitness-related component	Brain Regions (mm^3^)	Mathematics		Math calculation skills		Reading		Writing		Written expression		Science		Total achievement	
		b	*P*	b	*P*	b	*P*	b	*P*	b	*P*	b	*P*	b	*P*
**ActiveBrains**															
Cardiorespiratory fitness (mL/kg/min)	Inferior temporal gyrus	0.034	0.718	-0.006	0.950	-0.095	0.320	0.071	0.465	**0.207**	**0.030**	0.144	0.140	-0.010	0.913
	Inferior fronto-opercular gyrus	0.114	0.217	*0.163*	*0.079*	-0.014	0.886	0.030	0.756	0.036	0.710	0.091	0.350	0.035	0.705
Motor fitness (s^-1^)	Insular cortex	**0.199**	**0.031**	**0.258**	**0.005^∗^**	0.063	0.511	0.116	0.233	0.057	0.553	0.150	0.122	0.132	0.155
	Caudate	0.054	0.561	0.093	0.317	0.034	0.720	0.030	0.755	0.100	0.917	0.108	0.264	0.041	0.657
	Superior temporal gyrus	0.132	0.155	0.156	0.093	0.113	0.237	0.080	0.412	0.035	0.719	**0.194**	**0.044**	0.139	0.133
	Supramarginal gyrus	*0.155*	*0.094*	*0.154*	*0.098*	*0.174*	*0.065*	0.083	0.393	0.074	0.443	**0.208**	**0.031**	*0.166*	*0.071*
Muscular fitness (z-score)	Caudate	0.134	0.147	**0.189**	**0.040**	0.107	0.260	*0.164*	*0.087*	0.090	0.346	**0.228**	**0.017**	*0.151*	*0.100*
	Cerebellum IX	0.112	0.230	0.118	0.208	0.142	0.136	0.020	0.838	0.117	0.225	**0.199**	**0.040**	0.115	0.215
**FITKids2**															
Cardiorespiratory fitness (mL/kg/min)	Inferior temporal gyrus	0.114	0.485	-0.041	0.802	0.092	0.556	-0.043	0.780	-0.016	0.916	–	–	0.117	0.455
	Cingulate gyrus	-0.025	0.889	-0.113	0.521	0.003	0.986	-0.056	0.741	-0.069	0.677	–	–	0.031	0.857
	Middle Occipital Gyrus	0.153	0.365	0.061	0.715	0.070	0.664	0.041	0.799	0.016	0.919	–	–	0.112	0.490
	Fusiform Gyrus	0.192	0.237	0.136	0.398	0.094	0.545	-0.132	0.392	-0.142	0.348	–	–	0.086	0.585


*Values are standardized regression coefficients (β). Analyses were adjusted by sex, peak height velocity offset (years), parent education university level (neither/one/both) and body mass index (kg/m^2^). ^†^Academic performance was assessed by the Spanish version of the Woodcock-Johnson III in the ActiveBrains project and by the Kaufman Test of Educational Abilities in the FITKids2 project. Statistically significant values are shown in bold (P < 0.05), and borderline significant values are shown in italics (P < 0.1); ^∗^This was the only association that remained significant when P-values were adjusted for multiple comparisons using the Benjamini and Hochberg method to control for the false discovery rate.*

In the ActiveBrains project, among the brain regions previously associated with cardiorespiratory fitness, both regions were related or demonstrated a trend with an academic indicator; inferior temporal gyrus was related to written expression (β = 0.210; *P* = 0.030) and inferior fronto-opercular gyrus was marginally related to math calculation skills (β = 0.163; *P* = 0.079). Regarding the brain regions previously associated with motor fitness, insular cortex was related to mathematics and math calculations skills (β = 0.199 and β = 0.258, respectively; both *P* < 0.05); superior temporal gyrus and supramarginal gyrus were related to science (β = 0.194 and β = 0.208, respectively; both *P* < 0.05), and supramarginal gyrus was also marginally related to mathematics, math calculation skills, reading and total achievement (β ranging from 0.154 to 0.174; all *P* < 0.1). Regarding the brain regions previously associated with muscular fitness, caudate was related to math calculation skills and science (β = 0.189 and β = 0.228, respectively; both *P* < 0.05), and marginally related to writing and total achievement (β = 0.164 and β = 0.151, respectively; both *P* ≤ 0.01); cerebellum IX was related to science (β = 0.199; *P* = 0.040). However, after correcting for multiple comparisons, only the insular cortex remained significantly related to math calculations skills (β = 0.258; *P* < 0.005). In the FITKids2 project, none of the brain regions previously associated with cardiorespiratory fitness were related to academic performance.

## Discussion

The main finding of the present study is that cardiorespiratory fitness was positively related to white matter volume in overweight/obese children across two independent studies. In addition, other physical fitness components (i.e., motor and muscular) were also associated with white matter volume. Specifically, cardiorespiratory and motor fitness were related to white matter volume located in association fiber tracts, and muscular fitness was related to white matter regions located in thalamic radiations and projection fiber tracts. Moreover, some of these fitness-related associations in white matter volume were coupled with better academic performance. These results suggest that physical fitness might have the potential to enhance brain development and academic performance during childhood.

There are several possible explanations for the present findings. First, given that physical fitness has been previously related to gray matter in cortical and subcortical regions in children (Chaddock et al., 2010a,b; Esteban-Cornejo et al., 2017), it is reasonable that white matter structure connecting gray matter areas might also benefit from physical fitness. Indeed, white matter might be a neural mechanism via which physical fitness enhances integration of regions into networks and facilitates efficient transmission of information to support executive function and academic performance (Mabbott et al., 2006). Second, mouse models show that exercise, a major determinant of physical fitness, increases the number of oligodentrocytes, which are the cells responsible for myelinating axons in white matter tissue (Krityakiarana et al., 2010). Lastly, other biological mechanisms triggered by exercise, such as increased neurotrophic factors and vascularization, have been shown to influence white matter in rodents (Ding et al., 2006). Therefore, based on previous evidence from children and animal models, the positive associations between physical fitness and white matter found in overweight/obese children are neurologically and biologically plausible.

The major finding of the present report suggests that cardiorespiratory fitness may influence white matter volume in overweight/obese children across two independent, relatively large studies conducted in Spain and the United States. To note, although we focused on white matter volume, we superimposed the findings in a white matter tracts atlas for easier comparing and discussing present study with previous studies. Specifically, white matter brain regions that overlap between the ActiveBrains and FITKids project were located in the superior longitudinal fasciculus. Indeed, in the ActiveBrains project, all the white matter brain regions influenced by cardiorespiratory fitness were only located in the superior longitudinal fasciculus, whereas the white matter regions found in the FITKids2 project were located in the superior longitudinal fasciculus and the inferior longitudinal fasciculus. Both superior and inferior longitudinal fasciculi are long association fiber tracts that connect more distant cortical areas within the same cerebral hemisphere, converging in the parietal lobe, and may be involved in common pathways (Schmahmann et al., 2008). The superior longitudinal fasciculus links the frontal and parietal lobes, and may be engaged in attention, inhibition and articulatory aspects of language (Schmahmann et al., 2008; Chaddock-Heyman et al., 2013). The inferior longitudinal fasciculus links the parieto-occipital and temporal lobes, and has been shown to be engaged in object recognition, discrimination and memory (Schmahmann et al., 2008). Therefore, although only one brain region located in the superior longitudinal fasciculus overlapped between the ActiveBrains and FITKids2 projects, possibly due to methodological differences between studies (e.g., measurement of cardiorespiratory fitness, differences among the MRI scanners or the MRI sequence acquisition parameters, participant demographics, etc.), the fact that this finding was observed across the two different studies conducted on children strengthens conclusions regarding the specificity of the relations between cardiorespiratory fitness and white matter.

The analysis of the different components of physical fitness, assessed in the ActiveBrains project, allows us to further speculate on the relevance of each fitness component for white matter structure. Our data suggest that not only cardiorespiratory fitness, but also other components, such as motor and muscular fitness, were related to white matter volume among overweight/obese children. This effect in children supports and extends the associations between cardiorespiratory fitness and white matter in the older adults (Sexton et al., 2016). Among youth, there are only two studies examining the association between cardiorespiratory fitness and white matter structure in normal-weight youth and these studies exhibited contradictory findings (Chaddock-Heyman et al., 2014; Herting et al., 2014). Herting et al. (2014) revealed that cardiorespiratory fitness was negatively related to white matter microstructure in the corticospinal tract in male adolescents aged 15–18 years (Herting et al., 2014). In contrast, Chaddock-Heyman et al. (2014) found that cardiorespiratory fitness was positively related to the microstructure of white matter fiber tracts (i.e., body of the corpus callosum, superior corona radiata, and superior longitudinal fasciculus) in 9–10 year old children. The present findings comparing data from the ActiveBrains and FITKids2 project partially concur with the previous report from Chaddock-Heyman et al. (2014) since we found that the white matter brain regions predicted by cardiorespiratory fitness were mainly located in the superior longitudinal fasciculus and the inferior longitudinal fasciculus in overweight/obese children of the same approximate ages. However differences in white matter assessment (i.e., white matter volume vs. white matter microstructure) and in cardiorespiratory fitness levels (i.e., lower fit children vs. higher and lower fit children) among the present and the previous study should be acknowledged. Therefore, our findings shed light on the implications of cardiorespiratory fitness for brain health during childhood.

Specifically, the abovementioned cardiorespiratory fitness-white matter associations were only found in overweight/obese children, but not in their normal-weight peers. The reasons explaining why cardiorespiratory fitness might improve white matter volume only in overweight/obese children cannot be elucidated from the current datasets; yet two mechanisms might be speculated upon. First, moving an excessive amount of body mass is related to various musculoskeletal complaints associated with movement restrictions and motor difficulties during childhood (Paulis et al., 2014), which may have harmful implications for the musculoskeletal system, affecting skeletal neuromuscular function and the brain (Kullmann et al., 2015). For example, obese children have shown lower motor competence and white matter reduction in the cerebellar peduncles than their normal-weight peers (Augustijn et al., 2017). Experimental studies using mice exposed to prolonged movement restrictions has also shown negative effects on neurogenesis and the role of trophic determinants (i.e., nerve growth factor mRNA and brain-derived neurotrophic factor) involved in this phenomenon (Desaphy et al., 2005; Adami et al., 2018). Consequently, overweight/obese children may benefit more from increased cardiorespiratory fitness. Second, in overweight/obese children, not only is there an excess of body mass, but there is also an excess in a particular type of mass (i.e., fat mass vs. lean mass) which may have implications for maturation. That is, overweight/obese children demonstrate higher levels of fat mass than their normal-weight counterparts (Herda et al., 2018), which may confer additional white matter reductions (Kullmann et al., 2015). For example, when comparing correlations between total white matter volume and fat mass among normal-weight and overweight/obese children from the FITKids2 project, we found that fat mass was not associated with white matter in normal-weight children, but it was negatively associated with white matter in overweight/obese children. Therefore, the positive contributions of cardiorespiratory fitness to white matter might be more apparent in individuals with white matter reductions, such as overweight/obese children.

There is no previous evidence linking the other two physical fitness components, motor and muscular fitness, with white matter structure in youth, which hampers comparisons to other studies. The novel findings observed herein indicated that motor fitness was related to greater white matter volume in brain regions located in association fiber tracts (i.e., inferior fronto-occipital fasciculus and superior longitudinal fasciculus), and specifically, with larger white matter regions in the inferior fronto-occipital fasciculus. The inferior fronto-occipital fasciculus is the major white matter tract linking the ventrolateral and medial orbitofrontal cortices to the posterior parietal and occipital cortices. As such, greater white matter volume in brain regions located in this fasciculus could contribute to better prefrontal functioning, and in turn academic performance (Wu et al., 2016). Additionally, we found that muscular fitness was associated with greater white matter volume in brain regions mainly located in thalamic radiations (i.e., anterior thalamic radiation) and projection fiber tracts (i.e., middle cerebellar peduncle) in overweight/obese children. The anterior thalamic radiation is involved in reciprocal communication of limbic regions with prefrontal and anterior cingulate cortex, and the peduncle connects the cerebellum and other parts of the brain (Mori et al., 2005). Whereas these two tracts have been previously shown to be involved in higher-order motor tasks and influence cognitive inhibition (Glickstein and Doron, 2008; Chaddock-Heyman et al., 2013), we provide new support that muscular training aimed at improving upper- and lower-body muscular strength may influence white matter regions located in those motor tracts, and ultimately, academic performance during childhood. However, more research is warranted to understand how different components of physical fitness may effect white matter structures in both normal-weight and overweight/obese children.

Another interesting finding from the present study revealed that the white matter volume of regions related to fitness may influence academic performance. White matter helps enhance efficiency of neural transmission throughout the brain, and is thought to contribute to enhanced processing speed and executive function resulting in improvements of academic performance (Mabbott et al., 2006). In particular, a previous study in children aged 7–9 years suggested that microstructure in left white matter tracts (i.e., superior corona radiata and inferior longitudinal fasciculus) were related to better mathematical skills (van Eimeren et al., 2008). In addition, Li et al. (2013) showed that the inferior fronto-occipital fasciculus was related to better arithmetic scores. Consonant with those findings, we found that after correcting for multiple comparisons, motor fitness-related changes in white matter volume located in the left inferior fronto-occipital fasciculus was the only region related to better academic performance (i.e., math calculation skills) among overweight/obese children. As such, this finding must be interpreted with caution. Taken together, these findings raise the possibility that a reduction of physical activity opportunities across the school day might confer white matter reduction coupled with academic failure in children.

### Limitations and Strengths

Some limitations need to be considered. First, the cross-sectional design does not allow us to draw causal inferences, therefore these findings should be taken with caution; it is also possible that children with higher academic performance, had greater white matter volume and then performed better on physical fitness tests. Moving forward, it is important to replicate these preliminary findings using randomized controlled intervention studies. Second, since we approached the study with voxel-based morphometry, future studies should employ diffusion tensor imaging to examine white matter microstructure using a whole brain approach. Third, while both studies used a 3.0 Tesla Siemens Magnetom Tim Trio system, the head coils differed between studies, with a 32-channel head coil in the ActiveBrains study and a 12-channel head coil in the FITKids2 project. Previous studies have indeed shown signal-to-noise ratio improvements in the cortex for 32-channel head coils compared to 12-channel coils; these differences across coils are reversed for subcortical regions (Kaza et al., 2011). However, the fact that we observed a similar pattern of results across studies with different scan parameters could be seen as a strength as it speaks to the robustness of the observed effects. Because these and other methodological differences between the ActiveBrains and FITKids2 studies exist (e.g., assessment of cardiorespiratory fitness, the MRI sequence acquisition parameters, participant demographics, etc.), data from both projects were analyzed separately instead of pooling the datasets. While this approach limits the power of our findings, it offers replication across and the opportunity to qualitatively compare across studies. Lastly, some confounding variables that may influence the findings (e.g., diet, sleep, or self-discipline) were not available in both projects. Strengths of the present report include the use of data from two independent relatively large studies which speaks to the robustness of the observed findings, the complete and standardized assessment of the three physical fitness components, the whole-brain analysis, and the entire range of the BMI distribution among participants.

## Conclusion

In conclusion, our findings across two independent studies suggest that cardiorespiratory fitness may positively relate to white matter volume in overweight/obese children, and in turn, academic performance. In addition, other physical fitness components (i.e., motor and muscular) may also influence white matter volume coupled with better academic performance. Specifically, cardiorespiratory and motor fitness were related to white matter volume in brain regions located in association fiber tracts and muscular fitness was related to white matter regions located in thalamic radiations and projection fiber tracts. From a public health perspective, implementing exercise interventions that combine aerobic, motor and muscular training to enhance physical fitness may benefit brain development and academic success.

## Ethics Statement

This study was carried out in accordance with the recommendations of ‘name of guidelines, name of committee’ with written informed consent from all subjects. All subjects gave written informed consent in accordance with the Declaration of Helsinki. The protocol was approved by the Human Research Ethics Committee of the University of Granada and the Institutional Review Board of the University of Illinois at Urbana-Champaign.

## Author Contributions

IE-C had full access to all of the data in the studies. CH, FO, AK, KE, and AC conceived and designed the study. IE-C, LC-H, LR, CC-S, JM-G, and MR-A acquired the data. IE-C contributed to statistical analysis. IE-C, CS, MR-A, and JV-R interpreted the data. IE-C drafted the manuscript. All authors critically revised the manuscript for important intellectual content.

## Conflict of Interest Statement

The authors declare that the research was conducted in the absence of any commercial or financial relationships that could be construed as a potential conflict of interest.

## Footnotes

1 http://profith.ugr.es/activebrains

## References

[B1] Adami R. Pagano J. Colombo M. Platonova N. Recchia D. Chiaramonte R. (2018). Reduction of movement in neurological diseases: effects on neural stem cells characteristics. *Front Neurosci.* 12:336. 10.3389/fnins.2018.00336 29875623 PMC5974544

[B2] American College of Sports Medicine (2014). *ACSM’s Guidelines for Exercise Testingand Prescription*, 9th Edn Philadelphia, PA: Wolters Kluwer/Lippincott Williams & Wilkins.

[B3] Andersen S. L. (2003). Trajectories of brain development: point of vulnerability or window of opportunity? *Neurosci. Biobehav. Rev.* 27 3–18. 10.1016/S0149-7634(03)00005-8 12732219

[B4] Artero E. G. Espana-Romero V. Castro-Pinero J. Ruiz J. Jimenez-Pavon D. Aparicio V. (2012). Criterion-related validity of field-based muscular fitness tests in youth. *J. Sports Med. Phys. Fit.* 52 263–272. 22648464

[B5] Ashburner J. (2007). A fast diffeomorphic image registration algorithm. *Neuroimage* 38 95–113. 10.1016/j.neuroimage.2007.07.007 17761438

[B6] Ashburner J. Friston K. J. (2000). Voxel-based morphometry-the methods. *Neuroimage* 11(6 Pt 1), 805–821. 10.1016/j.neuroimage.2008.01.003 10860804

[B7] Ashburner J. Friston K. J. (2005). Unified segmentation. *Neuroimage* 26 839–851. 10.1016/j.neuroimage.2005.02.018 15955494

[B8] Augustijn M. Deconinck F. J. A. D’Hondt E. Van Acker L. De Guchtenaere A. Lenoir M. (2017). Reduced motor competence in children with obesity is associated with structural differences in the cerebellar peduncles. *Brain Imag. Behav.* 12 1000–1010. 10.1007/s11682-017-9760-5 28831722

[B9] Bar-Or O. (1983). in *Pediatric Sports Medicine for the Practitioner: From Physiologic Principles to Clinical Applications*, eds Katz M. Stiehm E. R. (New York, NY: Springer-Verlag). 10.1007/978-1-4612-5593-2

[B10] Benjamini Y. Hochberg Y. (1995). Controlling the false discovery rate: a practical and powerful approach to multiple testing. *J. R. Stat. Soc. Ser. B* 57 289–300. 10.1111/j.2517-6161.1995.tb02031.x

[B11] Cadenas-Sanchez C. Mora-Gonzalez J. Migueles J. H. Martin-Matillas M. Gomez-Vida J. Escolano-Margarit M. V. (2016). An exercise-based randomized controlled trial on brain, cognition, physical health and mental health in overweight/obese children (ActiveBrains project): rationale, design and methods. *Contemp. Clin. Trials* 47 315–324. 10.1016/j.cct.2016.02.007 26924671

[B12] Castro-Pinero J. Artero E. G. Espana-Romero V. Ortega F. B. Sjostrom M. Suni J. (2010a). Criterion-related validity of field-based fitness tests in youth: a systematic review. *Br. J. Sports Med.* 44 934–943. 10.1136/bjsm.2009.058321 19364756

[B13] Castro-Pinero J. Ortega F. B. Artero E. G. Girela-Rejon M. J. Mora J. Sjostrom M. (2010b). Assessing muscular strength in youth: usefulness of standing long jump as a general index of muscular fitness. *J. Strength Cond. Res.* 24 1810–1817. 10.1519/JSC.0b013e3181ddb03d 20555277

[B14] Chaddock L. Erickson K. I. Prakash R. S. Kim J. S. Voss M. W. Vanpatter M. (2010a). A neuroimaging investigation of the association between aerobic fitness, hippocampal volume, and memory performance in preadolescent children. *Brain Res.* 1358 172–183. 10.1016/j.brainres.2010.08.049 20735996 PMC3953557

[B15] Chaddock L. Erickson K. I. Prakash R. S. VanPatter M. Voss M. W. Pontifex M. B. (2010b). Basal ganglia volume is associated with aerobic fitness in preadolescent children. *Dev. Neurosci.* 32 249–256. 10.1159/000316648 20693803 PMC3696376

[B16] Chaddock-Heyman L. Erickson K. I. Holtrop J. L. Voss M. W. Pontifex M. B. Raine L. B. (2014). Aerobic fitness is associated with greater white matter integrity in children. *Front. Hum. Neurosci.* 8:584. 10.3389/fnhum.2014.00584 25191243 PMC4137385

[B17] Chaddock-Heyman L. Erickson K. I. Voss M. W. Powers J. P. Knecht A. M. Pontifex M. B. (2013). White matter microstructure is associated with cognitive control in children. *Biol. Psychol.* 94 109–115. 10.1016/j.biopsycho.2013.05.008 23714226 PMC3742734

[B18] Cole T. J. Lobstein T. (2012). Extended international (IOTF) body mass index cut-offs for thinness, overweight and obesity. *Pediatr. Obesity* 7 284–294. 10.1111/j.2047-6310.2012.00064.x 22715120

[B19] Desaphy J. F. Pierno S. Liantonio A. De Luca A. Didonna M. P. Frigeri A. (2005). Recovery of the soleus muscle after short- and long-term disuse induced by hindlimb unloading: effects on the electrical properties and myosin heavy chain profile. *Neurobiol. Dis.* 18 356–365. 10.1016/j.nbd.2004.09.016 15686964

[B20] Ding Y. H. Li J. Zhou Y. Rafols J. A. Clark J. C. Ding Y. (2006). Cerebral angiogenesis and expression of angiogenic factors in aging rats after exercise. *Curr. Neurovasc. Res.* 3 15–23. 10.2174/156720206775541787 16472122

[B21] Donnelly J. E. Hillman C. H. Castelli D. Etnier J. L. Lee S. Tomporowski P. (2016). Physical activity, fitness, cognitive function, and academic achievement in children: a systematic review. *Med. Sci. Sports Exerc.* 48 1197–1222. 10.1249/MSS.0000000000000901 27182986 PMC4874515

[B22] Espana-Romero V. Artero E. G. Santaliestra-Pasias A. M. Gutierrez A. Castillo M. J. Ruiz J. R. (2008). Hand span influences optimal grip span in boys and girls aged 6 to 12 years. *J. Hand Surg.* 33 378–384. 10.1016/j.jhsa.2007.11.013 18343294

[B23] Espana-Romero V. Ortega F. B. Vicente-Rodriguez G. Artero E. G. Rey J. P. Ruiz J. R. (2010). Elbow position affects handgrip strength in adolescents: validity and reliability of Jamar, DynEx, and TKK dynamometers. *J. Strength Cond. Res.* 24 272–277. 10.1519/JSC.0b013e3181b296a5 19966590

[B24] Esteban-Cornejo I. Cadenas-Sanchez C. Contreras-Rodriguez O. Verdejo-Roman J. Mora-Gonzalez J. Migueles J. H. (2017). A whole brain volumetric approach in overweight/obese children: examining the association with different physical fitness components and academic performance. The ActiveBrains project. *Neuroimage* 159 346–354. 10.1016/j.neuroimage.2017.08.011 28789992

[B25] Esteban-Cornejo I. Tejero-Gonzalez C. M. Martinez-Gomez D. del-Campo J. Gonzalez-Galo A. Padilla-Moledo C. (2014). Independent and combined influence of the components of physical fitness on academic performance in youth. *J. Pediatr.* 165 306.e2–312.e2. 10.1016/j.jpeds.2014.04.044 24952710

[B26] Freedson P. S. Goodman T. L. (1993). “Measurement of oxygen consumption. Measurement of oxygen consumption,” in *Pediatric Laboratory Exercise Testing: Clinical Guidelines*, ed. Rowland T. W. (Champaign, IL: Human Kinetics), 91–113.

[B27] Giedd J. N. Blumenthal J. Jeffries N. O. Castellanos F. X. Liu H. Zijdenbos A. (1999). Brain development during childhood and adolescence: a longitudinal MRI study. *Nat. Neurosci.* 2 861–863. 10.1038/13158 10491603

[B28] Glickstein M. Doron K. (2008). Cerebellum: connections and functions. *Cerebellum* 7 589–594. 10.1007/s12311-008-0074-4 19002543

[B29] Hayasaka S. Phan K. L. Liberzon I. Worsley K. J. Nichols T. E. (2004). Nonstationary cluster-size inference with random field and permutation methods. *Neuroimage* 22 676–687. 10.1016/j.neuroimage.2004.01.041 15193596

[B30] Herda T. J. Ryan E. D. Kohlmeier M. Trevino M. A. Gerstner G. R. Roelofs E. J. (2018). Examination of muscle morphology and neuromuscular function in normal weight and overfat children aged 7 to 10 years. *Scand. J. Med. Sci. Sports* 28 2310–2321. 10.1111/sms.13256 29959874

[B31] Herting M. M. Colby J. B. Sowell E. R. Nagel B. J. (2014). White matter connectivity and aerobic fitness in male adolescents. *Dev. Cogn. Neurosci.* 7 65–75. 10.1016/j.dcn.2013.11.003 24333926 PMC4020709

[B32] Huppertz C. Bartels M. de Geus E. J. van Beijsterveldt C. E. Rose R. J. Kaprio J. (2016). The effects of parental education on exercise behavior in childhood and youth: a study in Dutch and Finnish twins. *Scand. J. Med. Sci. Sports* 27 1143–1156. 10.1111/sms.12727 27455885 PMC5266726

[B33] Kail R. (1998). Speed of information processing in patients with multiple sclerosis. *J. Clin. Exp. Neuropsychol.* 20 98–106. 10.1076/jcen.20.1.98.1483 9672823

[B34] Kaza E. Klose U. Lotze M. (2011). Comparison of a 32-channel with a 12-channel head coil: are there relevant improvements for functional imaging? *J. Magn. Reson. Imag.* 34 173–183. 10.1002/jmri.22614 21618334

[B35] Krityakiarana W. Espinosa-Jeffrey A. Ghiani C. A. Zhao P. M. Topaldjikian N. Gomez-Pinilla F. (2010). Voluntary exercise increases oligodendrogenesis in spinal cord. *Int. J. Neurosci.* 120 280–290. 10.3109/00207450903222741 20374076 PMC2852894

[B36] Kullmann S. Schweizer F. Veit R. Fritsche A. Preissl H. (2015). Compromised white matter integrity in obesity. *Obesity Rev.* 16 273–281. 10.1111/obr.12248 25676886

[B37] Leger L. A. Mercier D. Gadoury C. Lambert J. (1988). The multistage 20 meter shuttle run test for aerobic fitness. *J. Sports Sci. Summer* 6 93–101. 10.1080/02640418808729800 3184250

[B38] Li Y. Hu Y. Wang Y. Weng J. Chen F. (2013). Individual structural differences in left inferior parietal area are associated with school childrens’ arithmetic scores. *Front. Hum. Neurosci.* 7:844. 10.3389/fnhum.2013.00844 24367320 PMC3854708

[B39] Mabbott D. J. Noseworthy M. Bouffet E. Laughlin S. Rockel C. (2006). White matter growth as a mechanism of cognitive development in children. *Neuroimage* 33 936–946. 10.1016/j.neuroimage.2006.07.024 16978884

[B40] Malina R. M. Rogol A. D. Cumming S. P. Coelho e Silva M. J. Figueiredo A. J. (2015). Biological maturation of youth athletes: assessment and implications. *Br. J. Sports Med.* 49 852–859. 10.1136/bjsports-2015-094623 26084525

[B41] McGrew K. Woodcock R. (2001). *Woodcock-Johnson III: Technical Manual.* Itasca, IL: Riverside Publishing Company.

[B42] Moore S. A. McKay H. A. Macdonald H. Nettlefold L. Baxter-Jones A. D. Cameron N. (2015). Enhancing a somatic maturity prediction model. *Med. Sci. Sports Exerc.* 47 1755–1764. 10.1249/MSS.0000000000000588 25423445

[B43] Mori S. Nagae-Poetscher L. M. van Zijl P. C. M. (2005). *MRI Atlas of Human White Matter.* Amsterdam: Elsevier BV.

[B44] Ortega F. B. Artero E. G. Ruiz J. R. Vicente-Rodriguez G. Bergman P. Hagstromer M. (2008a). Reliability of health-related physical fitness tests in European adolescents. The HELENA Study. *Int. J. Obesity* 32(Suppl. 5), S49–S57. 10.1038/ijo.2008.183 19011654

[B45] Ortega F. B. Ruiz J. R. Castillo M. J. Sjostrom M. (2008b). Physical fitness in childhood, and adolescence: a powerful marker of health. *Int. J. Obesity* 32 1–11. 10.1038/sj.ijo.0803774 18043605

[B46] Paulis W. D. Silva S. Koes B. W. Van Middelkoop M. (2014). Overweight and obesity are associated with musculoskeletal complaints as early as childhood: a systematic review. *Obesity Rev.* 15 52–67. 10.1111/obr.12067 23941399

[B47] Paus T. Collins D. L. Evans A. C. Leonard G. Pike B. Zijdenbos A. (2001). Maturation of white matter in the human brain: a review of magnetic resonance studies. *Brain Res. Bull.* 54 255–266. 10.1016/S0361-9230(00)00434-2 11287130

[B48] Raine L. Drollette E. Kao S. C. Westfall D. Chaddock-Heyman L. Kramer A. F. (2018). The associations between adiposity, cognitive function, and achievement in children. *Med. Sci. Sports Exerc.* 50 1868–1874. 10.1249/MSS.0000000000001650 29727406 PMC6095815

[B49] Ronan L. Alexander-Bloch A. F. Wagstyl K. Farooqi S. Brayne C. Tyler L. K. (2016). Obesity associated with increased brain age from midlife. *Neurobiol. Aging* 47 63–70. 10.1016/j.neurobiolaging.2016.07.010 27562529 PMC5082766

[B50] Ruiz J. R. Castro-Pinero J. Espana-Romero V. Artero E. G. Ortega F. B. Cuenca M. M. (2011). Field-based fitness assessment in young people: the ALPHA health-related fitness test battery for children and adolescents. *Br. J. Sports Med.* 45 518–524. 10.1136/bjsm.2010.075341 20961915

[B51] Schmahmann J. D. Smith E. E. Eichler F. S. Filley C. M. (2008). Cerebral white matter: neuroanatomy, clinical neurology, and neurobehavioral correlates. *Ann. N. Y. Acad. Sci.* 1142 266–309. 10.1196/annals.1444.017 18990132 PMC3753195

[B52] Sexton C. E. Betts J. F. Demnitz N. Dawes H. Ebmeier K. P. Johansen-Berg H. (2016). A systematic review of MRI studies examining the relationship between physical fitness and activity and the white matter of the ageing brain. *Neuroimage* 131 81–90. 10.1016/j.neuroimage.2015.09.071 26477656 PMC4851455

[B53] Song X. W. Dong Z. Y. Long X. Y. Li S. F. Zuo X. N. Zhu C. Z. (2011). REST: a toolkit for resting-state functional magnetic resonance imaging data processing. *PLoS One* 6:e25031. 10.1371/journal.pone.0025031 21949842 PMC3176805

[B54] Utter A. C. Robertson R. J. Nieman D. C. Kang J. (2002). Children’s OMNI scale of perceived exertion: walking/running evaluation. *Med. Sci. Sports Exerc.* 34 139–144. 10.1097/00005768-200201000-00021 11782659

[B55] van Bloemendaal L. Ijzerman R. G. Ten Kulve J. S. Barkhof F. Diamant M. Veltman D. J. (2016). Alterations in white matter volume and integrity in obesity and type 2 diabetes. *Metab. Brain Dis.* 31 621–629. 10.1007/s11011-016-9792-3 26815786 PMC4863900

[B56] van Eimeren L. Niogi S. N. McCandliss B. D. Holloway I. D. Ansari D. (2008). White matter microstructures underlying mathematical abilities in children. *Neuroreport* 19 1117–1121. 10.1097/WNR.0b013e328307f5c1 18596611

[B57] Vicente-Rodriguez G. Rey-Lopez J. P. Ruiz J. R. Jimenez-Pavon D. Bergman P. Ciarapica D. (2011). Interrater reliability and time measurement validity of speed-agility field tests in adolescents. *J. Strength Cond. Res.* 25 2059–2063. 10.1519/JSC.0b013e3181e742fe 21499136

[B58] Voss M. W. Chaddock L. Kim J. S. Vanpatter M. Pontifex M. B. Raine L. B. (2011). Aerobic fitness is associated with greater efficiency of the network underlying cognitive control in preadolescent children. *Neuroscience* 199 166–176. 10.1016/j.neuroscience.2011.10.009 22027235 PMC3237764

[B59] Wu Y. Sun D. Wang Y. Wang Y. (2016). Subcomponents and connectivity of the inferior fronto-occipital fasciculus revealed by diffusion spectrum imaging fiber tracking. *Front. Neuroanat.* 10:88. 10.3389/fnana.2016.00088 27721745 PMC5033953

